# Application of Novel Drugs for Corneal Cell Regeneration

**DOI:** 10.1155/2018/1215868

**Published:** 2018-05-02

**Authors:** Sang Beom Han, Yu-Chi Liu, Karim Mohamed-Noriega, Jodhbir S. Mehta

**Affiliations:** ^1^Department of Ophthalmology, Kangwon National University Hospital, Kangwon National University, Chuncheon, Republic of Korea; ^2^Singapore National Eye Centre, Singapore; ^3^Singapore Eye Research Institute, Singapore; ^4^Department of Ophthalmology, Yong Loo Lin School of Medicine, National University of Singapore, Singapore; ^5^Department of Ophthalmology, Faculty of Medicine, University Hospital “Jose E. Gonzalez”, Autonomous University of Nuevo Leon, Monterrey, NL, Mexico

## Abstract

Corneal transplantation has been the only treatment method for corneal blindness, which is the major cause of reversible blindness. However, despite the advancement of surgical techniques for corneal transplantation, demand for the surgery can never be met due to a global shortage of donor cornea. The development of bioengineering and pharmaceutical technology provided us with novel drugs and biomaterials that can be used for innovative treatment methods for corneal diseases. In this review, the authors will discuss the efficacy and safety of pharmacologic therapies, such as Rho-kinase (ROCK) inhibitors, blood-derived products, growth factors, and regenerating agent on corneal cell regeneration. The promising results of these agents suggest that these can be viable options for corneal reconstruction and visual rehabilitation.

## 1. Introduction

Corneal blindness is one of the leading causes of reversible blindness worldwide [1]. Corneal transplantation has been the only method for the treatment of corneal blindness, of which penetrating keratoplasty has long been the major surgical procedure. More recently, partial thickness corneal transplantation, such as Descemet's stripping endothelial keratoplasty (DSEK), Descemet's membrane endothelial keratoplasty (DMEK), and deep anterior lamellar keratoplasty (DALK), has been gaining popularity due to better visual prognosis and reduced risk of rejection. However, a global shortage of donor corneal tissue makes it impossible to meet the demands for corneal transplantation with current cornea donation programs. In addition, despite the advancement of surgical techniques and devices, corneal transplantation is still associated with limitations, such as graft failure or rejection, difficulty of the surgical procedure, and complications including secondary glaucoma [2, 3].

Remarkable developments of novel biomaterials and stem cell-based tissue cultivation and expansion techniques during the past few decades might enable the mass production of synthetic corneal tissue and are expected to provide innovative treatment methods for corneal disease. Medical treatment using drugs, such as Rho-kinase (ROCK) inhibitors, blood-derived products, growth factors, and regenerating agent eye drops (RGTA), is also anticipated to have therapeutic potential. In this review, we aimed to provide information on the pharmacologic therapies for corneal cell regeneration using these drugs.

## 2. Rock Inhibitors

ROCK is a serine/threonine protein kinase that is activated by Rho and forms Rho/ROCK complex that regulates a variety of cellular functions, such as cell proliferation, differentiation, migration, contraction, and apoptosis [4, 5]. Therefore, the ROCK signaling pathway has drawn interest as a potential target for the treatment of diseases of multiple organs [4, 5]. Recent studies showed that the ROCK inhibitor might be an innovative therapeutic agent for various ocular diseases, particular for corneal endothelial decompensation [4]. Although corneal transplantation has been the only therapy for corneal endothelial dysfunction, studies indicate the potential of ROCK inhibitors as a less-invasive alternative to graft surgery [3].

### 2.1. ROCK Inhibitor for Corneal Endothelial Cell (CEC) Generation

Okumura et al. [6] demonstrated that a selective ROCK inhibitor, Y-27632, promoted CEC proliferation and adhesion and suppressed its apoptosis, indicating that the topical ROCK inhibitor has been therapeutic for CEC regeneration [6]. An experimental study showed that a ROCK inhibitor facilitated the proliferation of CECs by the modulation of cyclin and p27; both are regulators of the G1/S transition [7]. Peh et al. [8] also revealed that Y-27632 increased cell proliferation. In their study, the effect of the ROCK inhibitor on the proliferation of CEC was significant only in the corneas from younger donors, suggesting that CECs from older corneas might lose the proliferation potential that could be activated by the ROCK inhibitor [8]. The findings of another study that Y-27632 had no effect on the proliferation of human CECs, although it facilitated corneal endothelial wound healing ex vivo and *in vitro*, support the assumption [9].

ROCK inhibitor eye drops enhanced corneal endothelial wound healing in a rabbit CEC injury model [6, 7, 10]. Topical ROCK inhibitor instillation also led to the facilitation of corneal endothelial wound healing and the recovery of corneal transparency in a primate CEC damage model [11]. Y-27632 was also shown to enhance the proliferation and adhesion and suppress the apoptosis of primate CECs cultured *in vitro* [6, 12]. Considering that primate CECs also have limited proliferative capacity, these results suggest that the ROCK inhibitor may have a therapeutic effect on human corneal endothelial dysfunction.

Okumura et al. [13] postulated that topical application of the ROCK inhibitor could facilitate the proliferation and migration of the residual CECs after acute corneal endothelial damage, thereby decreasing the risk of corneal endothelial decompensation [13]. A preliminary study showed that topical administration of the ROCK inhibitor resulted in the recovery of corneal transparency in 1-2 months in all 3 patients with postoperative acute corneal endothelial decompensation [3].

The ROCK inhibitor could be an alternative to corneal transplantation for Fuchs' endothelial corneal dystrophy (FECD). A human pilot study demonstrated that treatment with Y-27632 eye drops for patients with FECD resulted in the decrease in central corneal thickness and the recovery of corneal transparency in patients with corneal edema confined to the center, whereas the effect was not evident in those with diffuse corneal edema [3, 11, 14]. A recent case series study also revealed that descemetorhexis without graft surgery followed by topical ROCK inhibitor administration resulted in the restoration of corneal clarity and visual rehabilitation in patients with FECD [15].

### 2.2. ROCK Inhibitor for Tissue Engineering

For the treatment of corneal endothelial decompensation, the following two strategies for tissue engineering appears to be promising [1]: transplantation of bioengineered corneal endothelial cell sheet and [2] direct injection of cultivated CEC suspension to the anterior chamber [3].

Experimental studies showed that the transplantation of cultivated CECs on a collagen sheet can result in the restoration of corneal transparency and reduction of corneal edema [16, 17]. However, manipulation of the fragile monolayer sheet in the anterior chamber is technically challenging and is associated with the risk of CEC loss [3].

Theoretically, direct injection of CECs into the anterior chamber might be simple and less invasive compared to CEC sheet transplantation. In addition, the preparation of cells for transplantation may be easier, without the need for an artificial substrate [3]. The risk of CEC damage during the procedure might also be reduced. As the CECs injected into the anterior chamber may not spontaneously attach to Descemet's membrane, methods for improving the adhesion of the injected CECs have been attempted, such as magnetic guidance of iron powder or superparamagnetic microspheres incorporated in the CECs [18–20].

Considering that the ROCK inhibitor was proven to promote CEC adhesion onto a substrate [12], it may be postulated that the ROCK inhibitor can be helpful for promoting the attachment of CECs injected into the anterior chamber. An experimental study using a rabbit corneal endothelial dysfunction model demonstrated that intracameral injection of rabbit CECs combined with Y-27632, followed by keeping each rabbit in the facedown position for 3 hr, achieved enhanced attachment of donor cells onto host Descemet's membrane and the restoration of corneal transparency [21], whereas CEC injection without the inclusion of Y-27632 led to persistent corneal edema [21].

Y-27632 also promoted the adhesion of intracamerally injected CECs onto Descemet's membrane in both rabbit and primate corneal endothelial decompensation models and upregulated the expression of functional proteins including Na+/K+ ATPase and ZO-1, thereby leading to the resolution of corneal edema [22]. Another study revealed that intracameral injection of either human or monkey CECs in combination with Y-27632 led to the regeneration of monkey corneal endothelium, suggesting that intracameral injection of cultivated human CECs combined with the ROCK inhibitor may be a plausible therapeutic option for corneal endothelial diseases [2]. Preliminary data from a human clinical study showed that cultured human CECs injected in conjunction with ROCK achieved improvement of corneal edema and visual acuity without any serious adverse effect [3]. Although the results appear to be promising, further prospective randomized studies with a long-term follow-up are necessary to evaluate the efficacy and safety of ROCK inhibitors for the treatment of corneal endothelial diseases [3].

### 2.3. Other Effects of ROCK Inhibitor on Corneal Regeneration

The ROCK inhibitor was suggested to have additional effects that can be potentially applicable for corneal regeneration. Y-27632 was shown to promote both ex vivo and *in vitro* proliferation of limbal epithelial cell proliferation, suggesting it can be useful for the treatment of limbal stem cell deficiency [23]. Zhou et al. [24] also demonstrated that Y-27632 enhanced the cloning efficiency of limbal stem/progenitor cells by promoting their adhesion and capacity of reactive oxygen species scavenging in a rabbit model [24]. Y-27632 inhibited the transition of rabbit keratocyte to myofibroblast and modulated a wound healing process after a superficial lamellar keratectomy in a rabbit cornea [25]. Animal experimental studies revealed that ROCK inhibitors, fasudil and AMA0526, inhibited corneal neovascularization and opacity and facilitated corneal epithelial regeneration after corneal alkali burn [26, 27].

## 3. Blood-Derived Products

Application of blood-derived products for ocular surface diseases was first introduced in 1975 for 6 patients with chemical burns [28]. Since Tsubota et al. [29, 30] proved the efficacy and safety of autologous serum eye drops (ASE) for the treatment of dry eye disease (DED) and persistent epithelial defect (PED) [29, 30], studies have proven that blood-derived products are innovative therapeutic agents for various ocular surface disorders, such as DED [31], PED [32], neurotrophic keratitis [33], recurrent corneal erosion [34], and chemical burns [35].

### 3.1. Autologous Serum Eye Drops (ASE) [36]

ASE has a similar biochemical composition as human tears [37] and includes growth factors including epidermal growth factor (EGF) and transforming growth factor- (TGF-) *β* [38], chemokines, fibronectin, and various nutrients [38].

Consensus for the preparation method has never been established yet [37]. An *in vitro* experimental study indicated that an increased clotting time of 120 min or longer, a sharp centrifugation (3000*g* for 15 min), and dilution with balanced salt solution at 12.5–25% were optimal for corneal epithelial healing [39]. Clinically, 20% ASE is most frequently used to match the TGF-*β* concentration, which is 5 times higher in serum than in tears, to prevent delayed wound healing and promotion of corneal haze caused by TGF-*β* [30]. However, higher concentrations of ASE (50 to 100%) were also suggested to be effective and safe [40, 41]. A randomized prospective study showed that 100% ASE was more effective for the treatment of PED, Sjögren's syndrome (SS), and non-Sjögren DED than 50% ASE [42].

Randomized clinical studies revealed that ASE was more effective than conventional treatment for the improvement of both symptoms and signs of DED [43–45]. Kojima et al. [46] demonstrated that ASE suppressed apoptosis in the ocular surface epithelium and albumin contained in ASE recovered ocular surface damage [46]. ASE was superior to artificial tear in the improvement of dry eye signs after refractive surgery [47]. In addition, ASE can be a therapeutic option for PED refractory to conventional treatment [40, 48]. Schrader et al. [49] suggested that ASE combined with silicone hydrogel contact lenses might be helpful in recalcitrant PED [49]. Moreover, ASE was shown to be effective in facilitating reepithelialization of corneal graft after penetrating keratoplasty [50]. It also promoted corneal epithelial healing in patients with neurotrophic keratitis and aniridic keratopathy [51, 52]. A prospective study with a long-term follow-up revealed that ASE was effective for the prevention of recurrence in patients with recurrent corneal erosion [53]. However, a review of five randomized clinical trials that compared AS versus artificial tears or saline in DED patients revealed that no evidence of a benefit was found after two weeks of treatment, although there might be some short-term effect on symptoms with AS compared with artificial tears [54]. Therefore, we also believe further well-designed, large-scale randomized controlled studies are warranted to evaluate the efficacy of AS [54]. The absence of preservatives and high nutrient level in ASE increases the risk of sample contamination [55, 56]. Therefore, attention should be paid for the signs of infection in patients using ASE [36]. Lagnado et al. [55] recommended that sample vials should be stored frozen at −20°C for up to 6 months, and each vial should be thawed and used for only 24 hours. A prospective randomized human study showed that containers equipped with a sterilizing filter can be used for up to 4 weeks without any increased risk of contamination [56]. The concentrations of growth factors in 20% ASE remained stable for up to 9 months when kept frozen at −20°C and up to 4 weeks when defrosted [57].

### 3.2. Allogeneic Serum Eye Drops (SE)

Allogeneic SE from healthy donors can be advantageous in patients with fear or difficulty of blood sampling or coexisting blood disorders including anemia [58]. Moreover, it can also be a therapeutic option for patients with graft-versus-host disease (GVHD) or (SS), in which a considerable amount of proinflammatory cytokines could be included in their autologous serum [59]. Allogeneic SE can be prepared using the same protocol of ASE [58]. Because allogeneic SE includes anti-A and anti-B antibodies, it can theoretically cause an immune reaction against ABO antigens expressed on corneal and conjunctival epithelium [58]. Therefore, preparation of allogenic SE from ABO-identical donors or blood type AB donors is recommended [60, 61]. Studies demonstrated the efficacy of allogenic SE in DED, PED, neurotrophic keratitis, GVHD, and exposure keratopathy [60, 62, 63], indicating its potential as a viable alternative to ASE [36].

### 3.3. Umbilical Cord Serum Eye Drops (UCSE)

UCSE samples can be prepared using umbilical cord blood collected during delivery [36]. Rigorous screening for blood-borne infections is mandatory prior to donation [36, 58]. UCSE can also be a therapeutic option for patients with blood disorders or systemic inflammatory diseases, in which ASE is contraindicated [58]. Although allogeneic serum does have the same advantage, UCS contains a higher level of growth factors, neurotrophic factors, and essential tear components compared to allogeneic serum [31, 33, 64]. Moreover, compared to allogeneic serum, a substantially larger amount of UCSE can be obtained with a single sampling from one donor and can be distributed to multiple patients [58]. As UCSE contains high levels of neurotrophic factors such as substance P (SP), insulin-like growth factor- (IGF-) 1, and nerve growth factor (NGF) [32, 33], as well as growth factors including EGF and TGF-*β* [64], it is conceivably helpful for corneal nerve regeneration and epithelial healing. Studies have revealed that UCSE accelerated the recovery of PED and neurotrophic keratitis recalcitrant to conventional treatment [32, 33]. UCSE was shown to be more effective than ASE for the improvement of symptoms and signs of DED, particularly in severe cases associated with GVHD and SS [64, 65]. UCSE was superior to artificial tear in treating recurrent corneal erosions and reducing its recurrence [34]. In ocular chemical burn, UCSE resulted in faster corneal epithelial healing and milder corneal opacity compared to ASE or artificial tears [35]. UCSE is also shown to decrease early corneal haze and improve ocular surface parameters after laser epithelial keratomileusis (LASEK) [66].

### 3.4. Platelet-Derived Plasma Preparations

Platelet-derived plasma preparations contain a large amount of growth factors and cytokines [36, 58] and have been successfully used in maxillofacial and orthopedic surgery as well as in regenerative medicine for the promotion of tissue healing [67]. Various preparations have been developed, such as plasma rich in growth factors (PRGF), platelet-rich plasma (PRP), and platelet lysate [58]. PRGF is obtained by the filtration of plasma supernatants after centrifugation of the whole blood [68]. PRP is a plasma with increased concentrations of platelets obtained with an additional centrifugation of the whole blood [69]. Platelet lysate is collected by inducing platelet lysis and release of growth factors including platelet-derived growth factor (PDGF) using PRP [70].

Kim et al. [71] demonstrated that PRP was superior to ASE in the treatment of PED. PRGF was also suggested to be useful for the healing of PED [72]. PRP was shown to be effective for the improvement of both symptoms and signs of DED [73]. Plasma lysate was suggested to be helpful for the treatment of DED associated with GVHD or SS [74, 75]. PRP was also superior to conventional treatment for the recovery of visual acuity and corneal transparency in patients with ocular chemical injury [76]. In addition, PRP is potentially available for a biomaterial for ocular surface reconstruction [77, 78].

## 4. Growth Factors for Corneal Diseases

### 4.1. Nerve Growth Factor (NGF)

NGF facilitates corneal epithelial healing, which is mediated by the cleavage of *β*4 integrin and the upregulation of matrix metalloproteinase-9 [79]. Topical administration of NGF was shown to be effective in neurotrophic keratitis refractory to conventional treatment [80–82]. NGF is also expected to be effective for the treatment of diabetic keratopathy, as it could alleviate inflammation and apoptosis of corneal cells that can occur in diabetes mellitus [83].

Topical NGF was also shown to have a beneficial effect for postoperative corneal wound healing [80]. Cellini et al. [84] demonstrated that topical NGF was superior to artificial tear for corneal reconstruction after cataract. Animal experimental studies revealed that topical application of NGF accelerated restoration of corneal sensitivity and promoted cornea epithelial proliferation and nerve regeneration after laser in situ keratomileusis (LASIK) or photorefractive keratectomy (PRK) [85–87].

### 4.2. Substance P (SP) and Insulin-Like Growth Factor- (IGF-) 1

A randomized prospective study revealed that topical SP and IGF-1 combination therapy was useful for the prevention of superficial punctate keratopathy after cataract surgery in diabetic patients [88]. SP was shown to promote an epithelial healing process in diabetic cornea and attenuate hyperosmotic stress-induced apoptosis of corneal epithelial cells through the neurokinin-1 receptor signaling pathway [89, 90]. IGF-1 also facilitated the regeneration of corneal surface ultrastructure and nerves after LASIK in rabbit eyes [91].

### 4.3. Vascular Endothelial Growth Factor (VEGF)

VEGF can facilitate the functional and anatomical recovery after peripheral nerve damage [92]. Guaiquil et al. [93] demonstrated that VEGF-B treatment selectively promoted nerve regeneration and restored sensory and trophic functions of injured corneal nerves, suggesting that it might have a therapeutic potential for peripheral corneal nerve injury [93].

An experimental study revealed that the expression of endogenous VEGF-B was attenuated in regenerated corneal epithelium in a diabetic mouse model, whereas supplementation of exogenous VEGF-B accelerated corneal nerve regeneration [94].

### 4.4. Other Grow Factors

An animal study showed that pigment epithelial-derived factor, in conjunction with docosahexaenoic acid, might be effective for the treatment of DED caused by corneal nerve damage and neurotrophic keratitis [95]. Topical administration of neuroprotectin D1 was also shown to attenuate inflammation and facilitate nerve regeneration after corneal damage in a rabbit model [96]. Ciliary neurotrophic factor was shown to be able to activate corneal epithelial stem/progenitor cells and promote the corneal nerve regeneration and epithelial recovery, suggesting its therapeutic potential for diabetic keratopathy and limbal stem cell deficiency [97]. A randomized clinical study demonstrated that basic fibroblast growth factor promoted corneal epithelial healing after PRK, indicating it could be a therapeutic option for delayed healing [98].

### 4.5. Regenerating Agent Eye Drops (RGTAs)

RGTA (OTR4120 Cacicol20®; Théa, Clermont-Ferrand, France) is a carboxymethyl dextran sulfate polymer bioengineered to replace heparan sulfate, which is an important factor both for matrix proteins and for growth factors [99, 100]. Thus, RGTA is conceivably helpful for restoring equilibrium in cellular microenvironment [99, 101]. It is also expected to be useful for corneal wound healing, and several studies have shown promising results [99–107].

Experimental studies using a rabbit corneal burn model and a clinical case series study suggested that RGTA might be an innovative agent for promoting corneal regeneration and attenuating ocular surface inflammation by reducing oxidative, proteolytic, and nitrosative corneal damage [104, 108, 109]. A clinical pilot study also revealed the efficacy of RGTA for corneal ulcers and dystrophies refractory to conventional treatment [105]. A prospective clinical study demonstrated that RGTA might be effective and safe for the treatment of neurotrophic keratitis [106]. Chappelet et al. [101] recently showed that RGTA could be useful for the treatment of a PED after bacterial keratitis [101].

RGTA ophthalmic solution was also reported to facilitate corneal epithelial healing after corneal cross-linking (CXL) by reconstruction of the extracellular matrix in the corneal wound area [99, 100]. A randomized clinical trial demonstrated that RGTA might be superior to topical hyaluronic acid for corneal wound recovery after CXL in patients with keratoconus [103].

In animal excimer laser models, topical RGTA reduced corneal haze and promoted nerve regeneration, suggesting that it could be a useful option for the restoration of corneal microarchitecture after keratorefractive surgery [102, 107].

## 5. Conclusion

In this review paper, we have introduced a number of research papers that have demonstrated the efficacy of pharmacologic therapies, such as ROCK inhibitors, blood-derived products, growth factors, and RGTA on corneal cell regeneration. The promising results of these studies suggest that these agents can be viable options to aid corneal cell regeneration.

In summary, the ROCK inhibitor can promote the regeneration of CECs and its adhesion to Descemet's membrane. Thus, it can be used as a topical eye drops for the treatment of corneal endothelial dysfunction. It can also be used as an adjuvant therapy for CEC injection to AC. Blood-derived products can promote healing of ocular surface epithelium. Hence, it can be used for PED or neurotrophic keratitis. It can also be used for promoting ocular surface reconstruction and prevention of corneal opacity after ocular chemical injury. Growth factors promote the recovery of cornea epithelium and nerve; thus, these drugs can especially be helpful for PED, neurotrophic keratitis, and diabetic keratopathy. As RGTA facilitates corneal regeneration and attenuates ocular surface inflammation, it can be indicated for corneal regeneration after corneal CXL. It can also be helpful for the treatment of PDE or neurotrophic keratitis. The recovery of corneal epithelium and nerve after keratorefractive surgery can be facilitated by blood-derived products, growth factors, and RGTA (Table 1).

Although these therapeutic agents are innovative, further prospective randomized studies are needed for the verification of the efficacy and safety of the drugs [3]. Further studies are also required for the development of novel therapeutic agents for corneal cell regeneration.

## Figures and Tables

**Table 1:** Effect and possible application of the novel drugs.

Drug	Effect	Possible application
ROCK inhibitor	Corneal endothelial cell regeneration	Topical eye drops for recovery of corneal clarity in corneal endothelial dysfunction
	Promotion of corneal endothelial cell adhesion	Adjuvant therapy for the corneal endothelial cell injection

Blood-derived products	Promotion of healing of ocular surface epithelium	Recovery of persistent epithelial defect or neurotrophic keratitis
		Promoting ocular surface regeneration and prevention of corneal haze after ocular chemical injury or keratorefractive surgery

Growth factors	Facilitation of corneal epithelial healing and nerve regeneration	Treatment of persistent epithelial defect or neurotrophic keratitis
		Topical eye drops for diabetic keratopathy
		Recovery of cornea epithelium and nerve after keratorefractive surgery

RGTA^∗^	Promotion of corneal regeneration and attenuation of ocular surface inflammation	Treatment of persistent epithelial defect or neurotrophic keratitis
		Recovery of corneal epithelium after corneal cross-linking
		Corneal recovery after keratorefractive surgery

^∗^RGTA: regenerating agent eye drops.
